# Adjuvant Therapy Reduces Fat Mass Loss during Exercise Prescription in Breast Cancer Survivors

**DOI:** 10.3390/jfmk5030049

**Published:** 2020-07-15

**Authors:** Gabriele Mascherini, Benedetta Tosi, Chiara Giannelli, Elena Ermini, Leonardo Osti, Giorgio Galanti

**Affiliations:** Dipartimento di Medicina Sperimentale e Clinica, Università degli Studi di Firenze, 50134 Firenze, Italy; benedetta.tosi@unifi.it (B.T.); CHIARA_GIANNELLI@libero.it (C.G.); ermini.elena@gmail.com (E.E.); leo.osti@alice.it (L.O.); giorgio.galanti@unifi.it (G.G.)

**Keywords:** physical activity, oncology, unsupervised exercise, lifestyle, exercise prescription

## Abstract

Improvements in cancer care over the years have increased the numbers of cancer survivors. Therefore, quality of life, fat mass management and physical activity are growing areas of interest in these people. After the surgical removal of a breast cancer, adjuvant therapy remains anyway a common strategy. The aim of this study was to assess how adjuvant therapy can affect the effectiveness of an unsupervised exercise program. Forty-two women were enrolled (52.0 ± 10.1 years). Assessments performed at baseline and after six months of exercise prescription were body composition, health-related quality of life, aerobic capacity by Six-Minute Walk Test, limbs strength by hand grip and chair test and flexibility by sit and reach. Statistical analyses were conducted by ANOVA tests and multiple regression. Improvements in body composition, physical fitness and quality of life (physical functioning, general health, social functioning and mental health items) were found. The percentage change in fat mass has been associated with adjuvant cancer therapy (intercept = −0.016; b = 8.629; *p* < 0.05). An unsupervised exercise prescription program improves body composition, physical fitness and health-related quality of life in breast cancer survivors. Adjuvant therapy in cancer slows down the effectiveness of an exercise program in the loss of fat mass.

## 1. Introduction

Breast cancer is the most commonly diagnosed cancer in women around the world, the second cause of cancer death in female population of developed countries (198,000 deaths in 2012) and the first cause of cancer death in Italian women (12,274 deaths in 2015) [1].

The importance of lifestyle in the etiopathogenesis of this disease is well-demonstrated [2]. After a cancer diagnosis, patients report a feeling of fatigue that can result from the side effects of the treatment or from the cancer itself. This promotes an increase in physical inactivity, which increases the likelihood of incurring overweight and obesity [3]. The excess weight condition is associated with a low-grade systemic inflammation that promotes the development of insulin resistance, atherosclerosis and tumor growth, even in cancer survivors. This may explain the association between cancer and cardiovascular / metabolic diseases [4]. The impact of comorbidities on all-causes mortality in breast cancer survivors is remarkable [5].

Women with a diagnosis of breast cancer may experience disease and treatment-related adverse physiological and psychosocial effects at short and long-term [6]. After surgery, adjuvant therapy in the form of hormone therapy, chemotherapy or target molecular therapy is generally considered [1]. These therapeutic choices seem to promote short-term body composition changes by increasing body water and long-term in terms of increasing fat mass [7].

In order to reduce chronic inflammation, fat mass reduction is one of the most important outcome of any exercise prescription program, as it can contribute to decrease recurrence risk and to increase disease-free and overall survival [8]. Exercise training in breast cancer survivors can maintain or improve VO_2_peak, significantly improved lean body mass, upper and lower body strength [9].

Cancer can also negatively affect in terms of health-related quality of life (HRQoL) and psychosocial and physical function [6]. It is well known that physical activity has small-to-moderate beneficial effects on HRQoL, as well as on emotional or perceived physical and social function, anxiety, cardiorespiratory fitness [6,9] of breast cancers survivors during and after adjuvant treatment. HRQoL, whose improvements are considered a prognostic indicator of overall survival in cancer patients, normally worsens after cancer diagnosis and during cancer treatments [10,11].

Physical activity interventions may help to improve prognosis and may alleviate the adverse effects of adjuvant therapy in terms of body composition and HRQoL. Home-based exercise program demonstrated both a short and long-term effectiveness in physical function and body composition parameters [12,13].

This study aimed to:

- Evaluate how adjuvant therapy can influence the effectiveness of an unsupervised exercise program in terms of fat loss, analyzing how different therapeutic choices can have a different effect;
- Verify the effectiveness of an unsupervised exercise program on health-related quality of life in breast cancer survivors.

## 2. Materials and Methods

### 2.1. Subjects

The data of this observational study were collected from September 2015 to September 2017. The Breast Unit of the Careggi University Hospital selected and enrolled patients. After, they began the exercise prescription program at the Sports Medicine Center of the same University Hospital.

#### 2.1.1. Inclusion Criteria

The patients included in this study met the following inclusion criteria: (1) female, (2) from 21 to 65 years old, (3) physiological or pharmacological induced menopausal (4) history of surgery for breast cancer, (5) no participation in other training programs or no regular attendance at health clubs.

#### 2.1.2. Exclusion Criteria

The participants were excluded from the study if they said they were physically unable to participate in the treatment protocol or if changes in their physical activity behavior were contraindicated. The participants were also excluded if they were taking antipsychotic medication or undergoing any weight-loss strategy.

The participants were required to provide their written consent prior to their inclusion in the study as well as a letter of approval to participate from their oncologist and sports physician. All procedures performed in studies involving human participants were in accordance with the ethical standards of the institutional and national research committee and with the 1964 Helsinki declaration.

### 2.2. Procedures

The program at Sport Medicine Center consisted in:

- First visit (T0): history, cardiac evaluation, lifestyle assessment, body composition analysis, physical fitness parameters related to health and health-related quality of life.
- Follow-up visits (every 30 days): body composition analysis and health-related physical fitness parameters.
- six months follow-up visit (T5) body composition analysis, physical fitness parameters related to health and health-related quality of life.

#### 2.2.1. Medical History and Cardiac Evaluation

All patients were evaluated before starting the program to exclude any contraindication and thus provide eligibility to physical exercise. They underwent assessment by health questionnaire (to exclude any family history for chronic or metabolic diseases, anticancer therapies, comorbidity and any symptoms), physical examination, ECG at rest and 2-dimensional echocardiography to exclude chemotherapy-induced cardiotoxicity.

#### 2.2.2. Lifestyle Assessment

Lifestyle was assessed at the beginning of the program to evaluate the spontaneous physical activity [14]. An accelerometer (armband model MF-SW, display model DD100, SenseWear^®^, BodyMedia^®^, Pittsburgh, Pennsylvania, USA) on the non-dominant arm of the patients to be kept for one week. The parameters provided by the specific software were:

- Total energy expenditure in Kcal per day;
- Kcal > 3 METS expenses per day;
- PAL (physical activity level) defined as total energy expenditure/resting metabolic rate;
- Steps per day;
- Time spent in sedentary behaviors 1 to 1.49 METs (min);
- Light physical activity 1.5 to 2.99 metabolic equivalent of task (METs) mild physical activity (min);
- Moderate physical activity 3 to 5.99 METs (min);
- Vigorous physical activity> 6 METs (min);

#### 2.2.3. Body-Composition Analysis

The same researcher evaluated body composition, measuring anthropometric parameters, skinfold for subcutaneous adipose tissue and bio impedance for fat-free mass [15].

Measures of weight were approximated to the nearest 0.1 kg, those of height to the nearest 0.5 cm (Seca GmbH &Co., Hamburg, Germany), BMI was then calculated (kg/m^2^). Waist, hip, operated and not operated arm (in case of bilateral surgery, non-dominant arm values were considered as “operated”) circumferences were also measured using a measuring tape (Holtain Limited, Crosswell, UK, 1.5 m flexible tape). Waist–hip ratio was calculated [16].

Triceps, biceps, subscapular and supra-iliac skinfolds were measured by calipers (Holtain, Limited Tanner/Whitehouse skinfold caliper, Crosswell, UK) and sum (mm) of the four skinfold sites was calculated [17].

Bio impedance analysis (BIA 101 Sport edition, Akern, Florence, Italy) provided the values of resistance (Rz) and reactance (Xc) [18]: from these two the phase angle (PA), the amounts of total body water (TBW), extracellular water (ECW), intracellular water (ICW), fat free mass (FFM), body cell mass (BCM), muscle mass (MM) and fat mass (FM) were obtained.

#### 2.2.4. Health-Related Physical Fitness Parameters

The six minute walk test (6MWT) assessed cardiovascular fitness, because most daily life activities are performed at submaximal levels of exertion, this test may better reflect the functional exercise level for daily physical activities [19]. The parameters recorded during 6MWT were distance covered (6 MWD), peak heart rate with a heart rate monitor, systolic and diastolic blood pressure at rest and at the end of the test and self-perception of effort (CR10) [20].

Muscle fitness evaluation were performed with easily executable and reproducible tests in an outpatient setting such as sit & reach for flexibility [21], the hand grip test to estimate the overall static strength of the upper limbs with both arms [22] and the chair test to assess the strength of the lower limbs [23].

#### 2.2.5. Health-Related Quality of Life Assessment

Health-related quality of life was assessed by administering the SF-36 questionnaire. This is a validated tool that measures eight health concepts [24]: physical functioning (PF), role limitations due to physical health problems (RP), bodily pain (BP), general health (GH), vitality (VT), social functioning (SF), role limitations due to personal or emotional problems (RE) and mental health (MH) perceptions. Scores for each domain range from 0 to 100, with a higher score defining a more favorable health state [25].

#### 2.2.6. Exercise Prescription

Exercise program were prescribed at the end of the first visit following American College of Sports Medicine guidelines [26]. The program did not include supervised exercise. The combination of duration and weekly session aerobic training (such as walking, cycling or jogging) were established starting from thirty minutes five times per week (150 min per week) while intensity were establish in terms of heart rate and perceived of effort based on 6MWT. In addition, a target of number daily step were provided. At the end of each aerobic exercise sessions, flexibility exercise have been recommended. resistance training has been suggested twice per week with 8 exercises involving the main muscle groups, performed for 3 sets with 10 repetitions. The exercises were chosen based on the possibility of being performed safely at home (such as bodyweight squat and glute bridge for the lower limbs, lateral raise and biceps curl for the upper limbs). At the end of each visit, the prescribed exercise program was described. Furthermore, for resistance exercise, a demonstration was performed by qualified personnel in physical exercise followed by repetition by the patient as a learning test. Exercise program were individually updated every follow-up visit following the results of the assessments.

### 2.3. Statistical Analysis

The data were expressed as mean ± standard deviation. The Shapiro–Wilk test was used to assess the normal distribution of variables. one-way ANOVA was performed to evaluate the variations of the body composition and fitness parameters between baseline and final study values (T0-T5). The Cohen’s d effect size (ES) was calculated to determine the magnitude of effect. ES was assessed using the following criteria: small < 0.20, medium < 0.50 and large < 0.80.

The answers to the SF-36 questions were recorded and recalibrated to obtain a raw score that was then converted into the correspondent percentage score. A paired Student’s t-test was used to establish differences between baseline and six months SF-36 parameters.

A multiple linear regression was used to assess the relationship between the percentage change in fat mass during the program calculate as [(Δ T5-T0 FM/FM T0)·100)] and three potential predictors as: (1) adjuvant cancer therapy, (2) fat mass at baseline and (3) age at baseline.

One-way ANOVA and the Bonferroni’s test for multiple comparison were used to establish the potential differences among the different kinds of adjuvant therapy in term of change of fat mass. Therapy was considered with four subgroups: hormone therapy only, chemotherapy and/or target therapy, any combination of therapies, no therapy. The data were analyzed using SPSS-IBM 20 (SPSS, Inc., Chicago, IL, USA). The statistical significance threshold was set at a *p*-value = 0.05.

## 3. Results

A group of 42 women (age 52.0 ± 10.1 years) were considered eligible for the study and were then enrolled. All the patients had been diagnosed with a stage IIIC or inferior breast cancer and started the program after the surgery: 48% had undergone unilateral mastectomy with lymphadenectomy, 19% unilateral quadrantectomy with lymphadenectomy, 19% unilateral quadrantectomy, 12% bilateral mastectomy with bilateral lymphadenectomy, 2% bilateral mastectomy with unilateral lymphadenectomy.

Specific neoplastic therapy was: 15 took hormone therapy (tamoxifen and aromatase inhibitors), 9 underwent chemotherapy and/or target therapy (anthracycline and trastuzumab), 9 combined hormone therapy with chemotherapy and/or target therapy, and 9 did not undergo any adjuvant cancer therapy.

### 3.1. Lifestyle Assessment

The results of lifestyle assessment were:

- Total energy expenditure 2210.0 ± 336.0 kcal/day;
- Kcal> 3 METS 338.2 ± 263.6 kcal/day;
- Steps per day 8224.5 ± 2846.3;
- PAL 1.55 ± 0.18;
- Sedentary behaviors 16.5 ± 3.10 h/day;
- Light physical activity 4.9 ± 1.7 h/day;
- Moderate physical activity 78 ± 12.0 min/day;
- Vigorous physical activity 2.4 ± 0.02 min/day.

### 3.2. Body Composition Analysis

Baseline anthropometric parameters assessment defined an overweight sample (BMI T0 = 27.3 ± 4.20 kg/m^2^) and 30% was obese (BMI ≥ 30.0 kg/m^2^; Table 1).

During the six months of follow-up the patients lost weight progressively, getting close to overweight threshold (BMI T5 = 26.1 ± 3.87 kg/m^2^), others anthropometric parameters decreased, in particular waist circumference dropped below the cardio metabolic risk threshold. Skinfold thickness data and bio impedance analysis showed that weight loss is principally imputable to a fat mass loss and secondarily to extracellular water loss (Table 1).

On the contrary, body cellular mass and intracellular water did not show any significant change. The amount of total body water and fat free mass reduced is attributable to extracellular mass loss (Table 2).

### 3.3. Health-Related Physical Fitness Parameters

All the physical fitness parameters improved progressively. Moreover, the values at rest of systolic (SBP), diastolic (DBP) blood pressures and mean arterial pressure (MAP) decreased significantly (Table 3).

### 3.4. Health-Related Quality of Life Assessment

As far as the health-related quality of life is concerned, all the eight health concepts measured by the SF-36 questionnaire were improved: those found in PF, GH, SF and MH scales registered a significant improvement, and those found in BP, VT and RE scales were, anyhow, very close to significance threshold (Table 4).

### 3.5. Relationship between Adjuvant Cancer Therapy and Changes in Fat Mass

The association between percentage change in fat mass and the three potential predictors were (R^2^ = 0.52; MSE = 5.73): adjuvant cancer therapy coefficients = 8.63, *p* < 0.05; fat mass at baseline coefficients = −0.32, *p* = 0.36; age at baseline coefficients = 0.18, p = 0.26. In particular, this association between change in fat mass and adjuvant cancer therapy one-way ANOVA shows these differences existed between no therapy subgroup and any other subgroup (F = 5.12, *p* = 0.018, ES = 0.68), but no differences existed among these last subgroups of therapy:

- No therapy shows a fat mass reduction −16.5% ± 13.2%;
- Hormone therapy shows a fat mass reduction −6.5% ± 9.1%;
- Chemotherapy and/or target therapy shows a fat mass reduction −6.8% ± 10.4%;
- Hormone therapy + chemotherapy and/or target therapy shows a fat mass reduction −6.9% ± 9.0%.

Therefore, adjuvant cancer therapy was negatively associated with fat mass loss observed between baseline and six months.

## 4. Discussion

This study confirmed the effectiveness in terms of body composition and physical fitness of unsupervised exercise program in breast cancer survivor [12,13].

After the surgical removal of a breast cancer, adjuvant therapy is a common strategy. However, higher breast cancer risk with hormone replacement therapy is particularly evident among lean women, in postmenopausal women who are not taking exogenous hormones; general obesity is a significant predictor for breast cancer recurrence. Moreover, increased plasma cholesterol leads to accelerated tumor formation and exacerbates their aggressiveness [6].

The sample of the present study shows the anthropometric and lifestyle parameters in line with other studies already present [27]; these characteristics do not appear to guarantee a healthy level of cardiorespiratory fitness. Therefore, these patients should carry out a regular exercise program in order to ensure an improvement in health-related physical fitness parameters [28].

The therapeutic efficacy of the physical exercise is now consistent and demonstrated by systematic reviews and meta-analyses in the context of secondary and tertiary prevention of breast cancer [29,30]. Physical activity in breast cancer survivors may be more effective at modifying serum IGF-1 levels in women who are not taking tamoxifen [31], on the insulin pathway may be more pronounced for obese or sedentary women [32]. A marginal effect of physical activity in terms of decreasing circulating levels of biomarkers of inflammation in particular (CRP) [33] and in circulating levels of markers of cell-mediated immunity [34]. In this context, unsupervised training strategy could be an option in a physical exercise therapy perspective.

One of the aims of the study was to evaluate the influence of adjuvant therapy on the effectiveness of an exercise program on fat loss and how different therapeutic choices can have a different effect. A recent review [35] reports that exercise is effective in reducing fat mass during adjuvant therapy in breast cancer. However, the difference in efficacy between the different therapeutic strategies and the comparison of efficacy with those who do not perform any adjuvant therapy is not specified. The results of multiple regression in this study confirm the effectiveness of the exercise in reducing fat mass during adjuvant therapy without however any difference between the various therapeutic choices. However, those who did not have an adjuvant treatment regimen reported a greater reduction in fat mass than those who had an adjuvant therapy in place.

Health-related quality of life results are consistent with those shown in other studies where SF-36 questionnaire was used [36]. SF-36 is indeed a questionnaire that can be applied to many different clinical situations and that measures health concepts (particularly RE and MH) that can be influenced by a large number of factors (including changes in cancer therapies, that can signify changes in side effects associated with them). Anyway, the documented correlation between the other six scales and physical health perception, together with the improvements observed in body composition and physical fitness, justify the attribution to the program of at least a part of the improvements that is proportional to the correlation coefficient of each scale. Indeed, the lack of significance for RP, BP, VT and RE scales is almost surely due to the difference in statistical power that characterizes the eight scales: with a larger sample, significance would be obtained. Regarding RE and MH scales instead, it is only possible to consider that women have on average improved: this cannot be random, supporting the thesis that the program is capable of improving these two health concepts as well. It is important to notice that a major score in BP, RP and RE scales corresponds, respectively to minor pain, less limitation due to physical health problems and less limitation due to personal or emotional problems.

Poor prognosis in cancer survivors is associated with reduced levels of fitness, increased fat mass and decreased lean body mass [37]. Aerobic and resistance exercises prescription is capable of improving body composition, physical fitness and health-related quality of life of breast cancer survivors. American College of Sport Medicine’s guidelines [26] provide rough indications about intensity, duration and frequency of aerobic, resistance and flexibility exercises, highlighting the importance of considering a large number of factors (age, exercise endurance, drugs taken, cancer stage) to prescribe exercise safely and effectively. Anyhow, the ultimate goal of any exercise prescription program for cancer survivors is to induce long-term modifications in patients’ lifestyle, in order to reduce recurrence risk, cancer mortality and all causes of mortality [38]. From this perspective, unsupervised exercise programs can ensure good adherence in cancer survivors [39], even in those undergoing adjuvant treatments.

The present study shows strengths. First, all the subjects belonged to the same Breast Unit, therefore they undergone to surgery from the same surgeon group. Second, the sample receive the exercise program from the same Sport Medicine Center by the same specialist, therefore the methodology was standardized.

The study has limitations. The first limit is in the observational nature of an outpatient exercise prescription program; therefore, the intervention of the researcher was limited, and some adjustments were not possible. Second, body composition changes did not take into account the energy intake; therefore, the results have a reduced generalizability.

Larger samples are needed to confirm these results and future research directions could be the association between adjuvant therapies and the loss of fat mass assessed also with lipid blood values in addition to subcutaneous fat.

## 5. Conclusions

The results shown in this study demonstrate that an unsupervised exercised prescription program produces mid-term improvements in body composition, physical fitness and health-related quality of life of breast cancer survivors. Adjuvant therapy in cancer slows down the effectiveness of an exercise program in the loss of fat mass. Longer-term follow-up studies are needed to establish the real capacity of this training strategy to induce long-term lifestyle changes.

## Figures and Tables

**Table 1:** Anthropometrics and skinfold parameters during 6 months follow-up.

	T0	T5	Δ T5-T0	F Value	ANOVA	ES
Weight (kg)	71.9 ± 10.8	68.7 ± 10.1	−3.2 ± 2.3	6.47	<0.001	1.36
BMI (kg/m^2^)	27.3 ± 4.2	26.1 ± 3.9	−1.2 ± 0.9	6.12	<0.001	1.34
Waist circ. (cm)	90.2 ± 10.8	85.3 ± 9.8	−4.9 ± 4.0	6.02	<0.001	1.22
Hip circ. (cm)	106.1 ± 9.1	102.1 ± 7.1	−4.0 ± 3.9	5.76	<0.001	1.02
Waist/hip	0.85 ± 0.069	0.83 ± 0.06	−0.01 ± 0.04	0.23	NS	0.36
Operated arm circ. (cm)	31.8 ± 3.4	29.5 ± 2.6	−2.4 ± 1.7	5.43	<0.001	1.39
Not operated arm circ. (cm)	31.5 ± 3.4	29.4 ± 2.7	−2.1 ± 2.1	4.32	<0.001	0.99
Biceps skinfold (mm)	16.9 ± 8.1	13.8 ± 6.5	−3.1 ± 5.0	4.01	<0.001	0.61
Triceps skinfold (mm)	27.1 ± 5.7	23.5 ± 5.6	−3.6 ± 2.9	5.21	<0.001	1.26
Subscapular skinfold (mm)	25.5 ± 7.9	22.2 ± 7.1	−3.3 ± 3.9	4.55	<0.001	0.82
Supra−iliac skinfold (mm)	24.3 ± 8.9	21.0 ± 7.7	−3.3 ± 7.1	3.42	0.032	0.46
Skinfold sum (mm)	93.8 ± 25.5	80.5 ± 22.6	−13.3 ± 12.5	6.12	<0.001	1.06

**Table 2:** Bio-impedance parameters during 6 months follow-up.

	T0	T5	Δ T5-T0	F Value	ANOVA	ES
PA (°)	5.2 ± 0.7	5.3 ± 0.7	0.2 ± 0.7	0.31	NS	0.23
TBW (L)	35.0 ± 3.3	34.2 ± 3.3	−0.9 ± 1.8	3.12	0.025	0.48
ECW (L)	17.5 ± 1.9	16.8 ± 1.9	−0.7 ± 1.5	4.22	<0.001	0.50
ICW (L)	17.5 ± 2.3	17.4 ± 2.2	−0.1 ± 1.6	0.68	NS	0.08
FFM (kg)	46.7 ± 4.7	45.7 ± 4.4	−1.0 ± 2.9	4.88	0.002	0.36
BCM (kg)	23.1 ± 3.3	22.9 ± 3.1	−0.1 ± 2.1	1.08	NS	0.06
FM (kg)	25.0 ± 8.1	22.6 ± 7.2	−2.4 ± 3.4	4.23	<0.001	0.72

Legend: PA—phase angle; TBW—total body water; ECW—extra cellular water; ICW—intra cellular water; FFM—fat free mass; BCM—body cellular mass; FM—fat mass.

**Table 3:** Physical fitness parameters related to health during 6 months follow-up.

	T0	T5	Δ T5-T0	F Value	ANOVA	ES
Chair test (reps)	14.5 ± 3.8	18.3 ± 4.3	3.8 ± 2.6	8.12	<0.001	1.45
Hand Gr. op. arm (kg)	24.3 ± 4.8	26.5 ± 4.5	2.2 ± 4.5	3.55	0.012	0.48
Hand Gr. not op. arm (kg)	24.2 ± 4.6	26.4 ± 4.3	2.2 ± 3.1	4.78	<0.001	0.72
Sit and reach test (cm)	2.6 ± 9.3	8.5 ± 7.1	5.8 ± 6.0	6.02	<0.001	0.97
6 MWD (m)	518.6 ± 133.0	584.8 ± 97.2	66.2 ± 107.2	4.28	<0.001	0.62
HR rest (bpm)	75.6 ± 13.5	73.5 ± 10.5	−2.1 ± 11.8	3.92	0.015	0.18
SBP rest (mmHg)	117 ± 15.1	110 ± 12.7	−6.4 ± 13.4	3.04	0.048	0.48
DBP rest (mmHg)	76.1 ± 11.3	70.6 ± 8.7	−5.5 ± 9.70	4.11	0.013	0.57
MAP rest (mmHg)	89.7 ± 11.5	83.9 ± 9.2	−5.8 ± 9.8	4.11	0.012	0.59

Legend: HR—heart rate; SBP—systolic blood pressure; DBP—diastolic blood pressure; MAP—mean arterial pressure.

**Table 4:** Results of health-related quality of life parameters measured by social functioning (SF)-36 questionnaire.

	T0	T5	Δ T5-T0	*p*-Value
PF (%)	72.7 ± 24.6	83.7 ± 17.1	11.0 ± 15.9	<0.001
RP (%)	61.3 ± 39.1	65.5 ± 37.4	4.17 ± 33.5	0.43
BP (%)	61.0 ± 25.8	67.4 ± 22.0	6.40 ± 30.3	0.18
GH (%)	64.7 ± 20.4	69.1 ± 18.9	4.43 ± 6.97	<0.001
VT (%)	52.7 ± 18.4	57.4 ± 16.7	4.64 ± 15.0	0.051
SF (%)	60.5 ± 24.5	67.6 ± 22.9	7.08 ± 20.1	0.027
RE (%)	56.4 ± 43.2	65.9 ± 33.3	9.53 ± 37.7	0.11
MH (%)	63.4 ± 14.8	67.3 ± 12.5	3.90 ± 10.7	0.022

Legend: PF—physical functioning; RP—role limitations due to physical health problems; BP—bodily pain; GH—general health; VT—vitality; SF—social functioning; RE—role limitations due to personal or emotional problems; MH—mental health perceptions.
